# The mediating roles of psychological resilience and frustration tolerance in the relationship between coping styles and mood states of high-level basketball referees

**DOI:** 10.3389/fpsyg.2023.1096649

**Published:** 2023-06-07

**Authors:** Qiulin Wang, Wei Wang, Huanhuan Huang, Binghui Wan

**Affiliations:** College of Physical Education, Yangzhou University, Yangzhou, Jiangsu, China

**Keywords:** coping style, mood state, psychological resilience, frustration tolerance, basketball referee

## Abstract

**Objective:**

In order to promote the development of high-quality professional basketball referees in China, we explored the relationship between their coping styles and mood states during the game and assessed the contributions of psychological resilience and frustration tolerance to this relationship.

**Methods:**

A total of 364 national and international male and female basketball referees were recruited and surveyed *via* the online questionnaire platform “Questionnaire Star”. All participants signed an informed consent form and completed the questionnaire. Common method bias test and Pearson correlation tests were used to analyze the study indicators, and the theoretical model for this study was validated using Process plug-in developed by Hayes.

**Results:**

The results of the study showed that the coping style of the referees significantly predicted their psychological resilience, frustration tolerance, and mood state. Coping style enhanced psychological resilience (*β* = −0.30) and frustration tolerance (*β* = 0.38) and improved the mood states (*β* = 0.33) of the referees. In addition, coping style directly predicted mood state but also indirectly predict mood state through the intermediary variables of psychological resilience (*β* = 0.14) and frustration tolerance (*β* = 0.11), and the mediating effects accounted for 24.20 and 18.90% of the total effect, with psychological resilience playing a greater role than frustration tolerance. (β: standardized regression coefficient).

**Conclusion:**

These findings suggest that when training high-level basketball referees, increasing the psychological indicators related to the coping styles and psychological resilience of high-level basketball referees can avoid their large emotional fluctuations and improve their accuracy in judging when facing unexpected events on the court.

## Introduction

### Mood state

In basketball matches, referees’ calls are the result of cognitive decision-making processes in which the referee (observer) judges the behavior of the players (observed). Given that the referee can be considered the subject and the athlete considered the object, the referee’s decision-making behavior is affected by the subject, object, and the interactive environment between the subject and object (i.e., the referee, the athlete, and the competition environment). Sports competitions provide people with a source of tension and stimulation as staged, planned social conflicts. Sports spectators who participate in the event through their own perceptions release their emotions in both positive and negative ways. Thus, during competition, spectators may show antagonistic or anti-hierarchical emotional characteristics. They may not accept the decision of the referee silently but instead show direct emotional reactions, which may include poor behavior, such as loudly voicing criticism or even abusing the referee (Tao, 2016). Referees who are affected by these emotional displays may experience mood swings. Previous studies have assessed the ability of referees to perform their job based on their personal qualities. With the development of referee discretion and audience marketing of spectator sports in China, the importance of the referee’s psychological qualities has become particularly prominent.

The relationship between emotion and cognition is a consistent focus in the field of emotional psychology research. From Schacht’s emotional cognition theory to the positive emotion expansion theory of positive psychology, all research findings indicate that emotion influences cognition. In the past, research assessing emotion and cognitive decision-making in the field of competitive sports was mainly conducted with athletes. However, referees, an indispensable part of the modern competitive arena, are also under tremendous pressure, especially at critical moments in competition; thus the pressure on referees is no less than that on athletes. Therefore, the emotional state of referees will inevitably affect their cognition and behavior, in this case their decision-making abilities. Mood states (i.e., persistent and weak emotional states) exacerbate an individual’s emotional experience (Wang et al., 2021), and a change in a mood state affects an individual’s movement or behavior (Zhu, 1995). There is a high correlation between mood state and a referee’s on-the-spot performance (Pizzera et al., 2022). Unstable mood states adversely affect a referee’s decision-making ability (Brandão et al., 2014). The work of basketball referees requires comprehensive abilities, including the understanding of the theories and rules underlying the sport and their physical abilities, psychological qualities, and the ability to make accurate immediate judgment calls. Among them, strong psychological qualities help a referee to maintain a good mood state in the game and thus maintain better on-the-spot judgment calls (Wang, 2017). Therefore, it is of great practical significance to assess mood states of basketball referees and the related variables that affect their mood states.

### Coping style

Coping style refers to the way in which people use conscious and behavioral efforts to evaluate their own abilities and to reduce internal and external pressure. There is a correlation between coping style and mood state. Previous studies have shown that coping style directly affects depression (Zhang et al., 2005); negative avoidance coping is positively correlated with depression, whereas positive action coping is negatively correlated with depression (Niu et al., 2013). The interaction model of coping asserts that the choice of an individual’s coping style is the result of interactions among personality traits, individual differences, and environmental stress. The factors influencing coping styles are generally divided into stability factors and situational factors. Stability factors include an individual’s gender, age, personality traits, and the like. However, the influence of personality factors on coping styles is restricted by situational factors, which mainly include the objective characteristics of the stressful situation (such as the degree of stress, the degree of controllability, and the variability of the situation) and the subjective understanding and evaluation of the situation by individuals. According to the classification of coping styles from the perspective of coping function, there are general functional dimensions in individual coping styles. Individuals take these general functional dimensions as a starting point and then combine their own coping resources, situational characteristics, and other factors to establish their own coping styles (Ye and Shen, 2002). However, situations in high-level basketball matches are highly variable. As the executor of the rules of the game, the referee needs to combine rich experience in refereeing with reasonable and accurate judgments of complex situations to establish an immediate response when facing complex and changing game situations. Research assessing coping styles and improving an individual’s psychological and mental states shows that higher positive coping scores are associated with better mood states, whereas higher negative coping scores are associated with worse mood states (Anshel et al., 2014). Wang and Jiang (2018) found that football referees’ coping styles of facing and yielding are negatively correlated with their negative mood state, but avoiding is positively correlated with a negative mood state. Therefore, a positive coping style is a psychological quality that a referee must possess to maintain a good mood state and carry out effective refereeing. Given that coping style is related to the mood state of basketball referees when they make decisions on the spot, which is directly related to the accuracy of referees’ decisions. We hypothesized that: The coping style of basketball referees would positively predict mood state.

### Psychological resilience

Psychological resilience generally refers to the ability of an individual to recover quickly after experiencing setbacks and to develop corresponding coping styles with constant repetition so that the individual becomes increasingly better at handling setbacks and thus achieves growth (Wang and Jiang, 2018). Onwukwe (2010) found that a positive coping style is a protective factor in psychological resilience. Research assessing psychological resilience across different populations, ages, occupations, social strata, and physical health conditions has shown a positive correlation between positive coping styles and psychological resilience. Studies examining the relationship between psychological resilience and mood state have found that psychological resilience improves mood state and promotes mental health (Li and Li, 2014). Compared with professional referees, amateur referees are more likely to be affected by all aspects of stress, whereas professional referees have more reasonable coping strategies to deal with their emotions. As an important psychological behavior in the process of self-regulation, coping style is a protective factor to promote individual psychological resilience. Individuals with high psychological resilience show a high degree of adaptability and better mood states (Han and Wang, 2022). Thus, the second hypothesis of this study was that: Psychological resilience would play a mediating role between coping styles and mood states of high-level basketball referees.

### Frustration tolerance

Frustration tolerance refers to the extent to which an individual accepts setbacks. It is one of the most basic internal qualities of human psychology, an essential factor in a person’s personality structure, and a core part of a person’s self-expression consciousness (Luo and Zhou, 2015). The concept of frustration tolerance is similar to that of psychological resilience, but there are some differences. Whereas frustration tolerance is an essential factor in personality structure, psychological resilience is a factor in personality traits (Friborg et al., 2005). It is inevitable that referees will be condemned by others during their career and will make mistakes in judgment, which may lead to self-blame, regret, and frustration. In addition, extreme behaviors by coaches, athletes, or spectators may lead to referees having negative emotions and thus affect their ability to referee (Lin, 2008). When an individual experiences a setback, whether caused by external or internal factors, the setback will be accompanied by complex emotional reactions, such as anxiety, tension, worry, unease, fear, depression, and anger, as well as an imbalance in psychological and physiological activities (Li et al., 2020). Individuals with poor tolerance are more likely to adopt negative coping styles, whereas individuals with strong tolerance are more likely to adopt positive coping styles (Li and Li, 2014; He and Chen, 2021). Frustration may enhance tolerance to setbacks, improve the ability to withstand setbacks, and enable individuals to maintain good mood states when in a state of high tension, emotional depression, sleeplessness, hunger, or anxiety. Therefore, this study’s third hypothesis was that: Frustration tolerance would play a mediating role between coping styles and mood states in high-level basketball referees.

Therefore, this study investigated whether the coping style of high-level basketball referees positively predicted their mood state and whether psychological resilience or frustration tolerance played a mediating role between coping styles and mood states.

## Materials and methods

**Figure 1:**
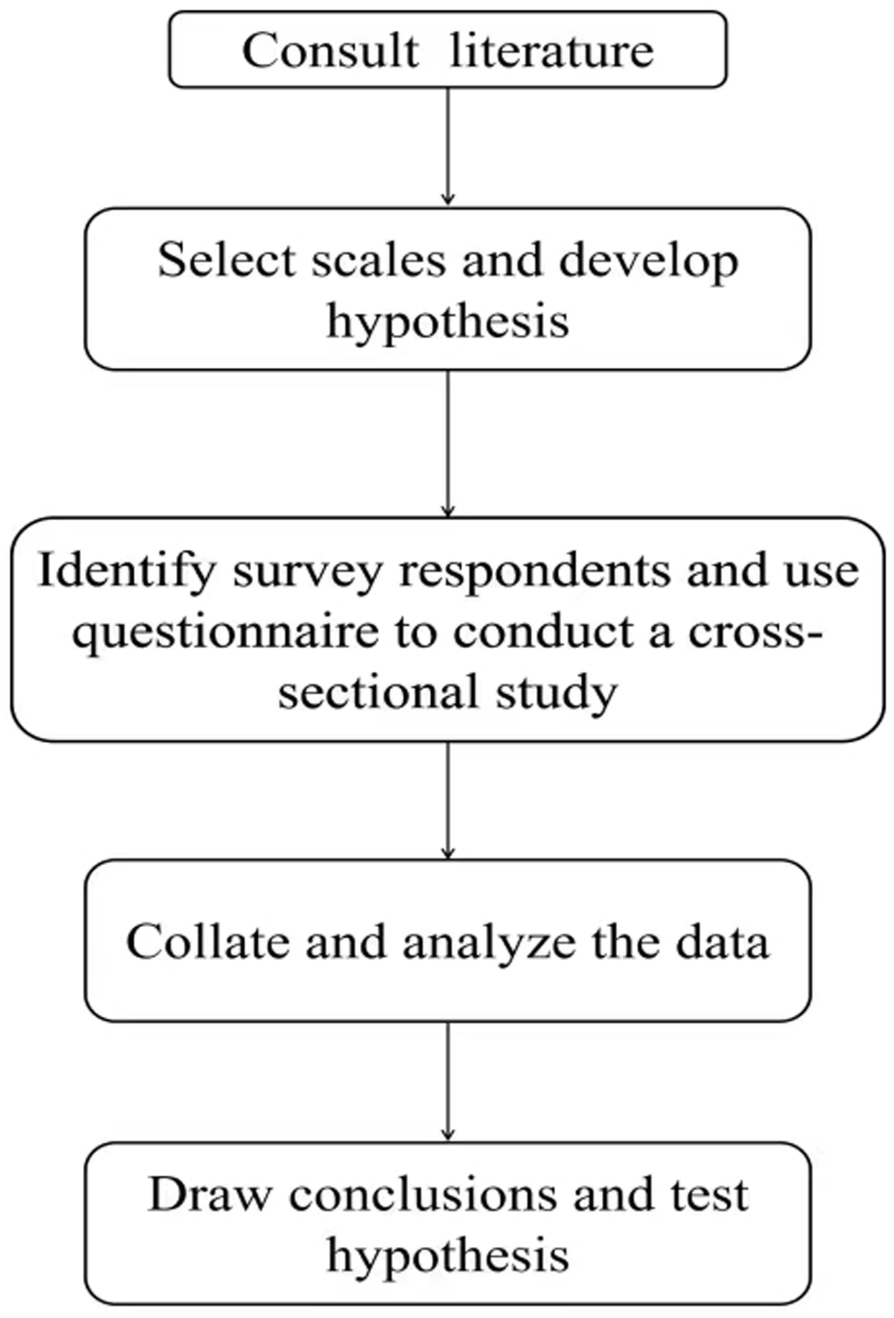
Research structure diagram.

This study was approved by the Ethics Committee of Yangzhou University Medicine College (approval No. YXYLL-2022-126). All participants provided written informed consent prior to answering questionnaires. As for the specific research structure diagram, see Figure 1.

### Participants

In total, 364 national and international basketball referees (37.73 ± 6.91 years) from basketball leagues of all levels in the 2021–2022 season participated in this study. The referees worked in first-tier professional basketball leagues in China, including the Chinese Basketball Association (CBA), the Women’s Chinese Basketball Association (WCBA), the National Basketball League (NBL), and other leagues, which included the Chinese University Basketball Association (CUBA) and the Women’s Chinese University Basketball Association (WCUBA). At present, they are the highest-level representatives of basketball referees in China. See Table 1 for referee demographic characteristics and league-related information.

**Table 1:** Demographic and league information for 364 basketball referees who participated in this study.

Variable	Category	Number of referees	Sanction time (years)	Average age (years)
Gender	Male	312	3.22	37.87
	Female	52	4.48	36.79
Type of league	CBA	48	8.1	41.29
	WCBA	98	5.0	37.49
	NBL	36	2.83	37.33
	Other	182	1.41	36.97
Referee grade	International	29	8.41	37.55
	National	335	2.96	37.73

### Measures

All scales and questionnaires used in the study were distributed through the website Questionnaire star. Questionnaire Star is an online platform that distributes questionnaires for individuals to complete and provides an informed consent form for individuals to sign. A total of 450 sets were distributed and 425 sets were recovered, leading to a recovery rate of 94.44%. All participants e-signed an informed consent form and completed the questionnaire. Participants filled in each set of four questionnaires according to the stated guidelines. Questionnaires were evaluated to ensure they met pre-study set standards, and we removed 86 invalid questionnaires after screening. This left a total effective sample size of 364 complete sets (85.6% of the recovered total). The scales used in this study have been widely used in many studies and have good reliability and validity.

#### Psychological resilience scale

We used the psychological resilience scale originally developed by Connor and Davidson (2003) and modified for Chinese participants (Yu and Zhang, 2007). There were 25 items that assessed tenacity, psychological strength, and optimism. Each item was rated by the participant as 0 to 4 points (5-point scale). The higher the score, the higher the individual’s psychological resilience level. In this study, Cronbach’s α was 0.952, and the Kaiser-Meyer-Olkin (KMO) value was 0.962, indicating that the scale had high reliability and validity.

#### Coping style scale

We used the Coping Styles Questionnaire, which is composed of 62 items with six subscales: avoidance, fantasy, self-blame, help-seeking, rationalization, and problem solving (Zhang et al., 2021). Each item was answered by the participant as “yes” (1 point) or “no” (0 points). When participants chose *yes*, they were asked to evaluate the effectiveness by selecting “effective,” “relatively effective,” and “invalid.” The higher the score, the more inclined the participant was to adopt a certain coping style. In this study, Cronbach’s α was 0.891, and the KMO value was 0.888, indicating that the scale had high reliability and validity.

#### Frustration tolerance scale

We used the Chinese revised version of the Frustration Tolerance questionnaire (Wang et al., 2014), which comprised 28 items with 4 dimensions: avoidance, difficulty, power, emotional tolerance, and achievement. Each item was rated by the participant on a 5-point Likert scale. Higher scores indicated worse frustration tolerance. In this study, Cronbach’s α was 0.952, and the KMO value was 0.949, indicating that the scale had high reliability and validity.

#### Mood state scale

Mood state was measured using the Mood State Scale developed by Zhu (1995). In total, 40 items assessed 7 dimensions of mood states, including tension, anger, fatigue, depression, panic, energy, and self-esteem. Each item was rated by the participant on a 5-point Likert scale. Higher total scores indicated greater negative emotional states, that is, the more upset or maladjusted the mood (the higher the score, the worse the mood). In this study, the overall Cronbach’s α was 0.937, and its subscale Cronbach’s α were 0.850, 0.906, 0.859, 0.905, 0.855, 0.904, and 0.727. The KMO value was 0.961, indicating that the scale had high reliability and validity. (The total score is equal to the score of the five negative scales minus the score of the two positive scales plus one hundred).

### Statistical analysis

We used SPSS 26.0 for statistical analysis. *T*-tests and analyses of variance were used as appropriate to assess the differences among referees at all levels and among league variables. Pearson correlation analysis and regression analysis were conducted among the variables. We use the SPSS macro program Process plug-in compiled by Hayes for mediation analyses and bootstrap analyses. In the statistical analysis of the data, we set *p* < 0.05 as the significant level, and *Cohen’s d* = 0.2 (*η*^2^ = 0.01), *Cohen’s d* = 0.5 (*η*^2^ = 0.059), and *Cohen’s d* = 0.8 (*η*^2^ = 0.138) correspond to small, medium and large effect sizes respectively (Hu, 2010).

## Results

### Assessment of common method bias

Because the measurement method in this study comprised only questionnaire surveys, we used the Harman single-factor test to assess common method bias. The results show that the number of common factors for extracting feature roots >1 was 32, and the first common factor explained 18.732% of the total variation. This value was less than the 40% threshold standard, indicating that there was no serious common method bias.

### Psychological differences between referees in national versus international leagues and across leagues

Independent sample *T*-tests were used to assess differences in coping style, mood state, psychological resilience, and frustration tolerance of basketball referees working at the national vs. international level. The results indicated that psychological resilience among international referees was better than that among national referees (*t* = 2.571, *p* < 0.05, *Cohen’s d* = 0.56), but there was no significant difference in coping styles, mood state, or frustration tolerance between the international and national referees (Table 2).

**Table 2:** Coping Style, mood state, psychological resilience and frustration tolerance of referees at national vs. international levels.

Variable	International level (*n* = 29)	National level (*n* = 335)	*t*	*p*
	Mean ± SD score	Mean ± SD score		
Psychological resilience	76.17 ± 11.31	68.16 ± 16.44	2.571	0.011
Coping style	29.52 ± 8.29	32.1 ± 9.76	−1.381	0.168
Mood state	103.86 ± 21.56	111.28 ± 20.72	−1.843	0.066
Frustration tolerance	82.28 ± 17.02	80.08 ± 18.41	0.619	0.536

Single factor analysis of variance was used to compare the differences in psychological resilience, coping style, mood state, and frustration tolerance of referees in different types of competitions. The results showed that mood state for referees in the CBA league was the same as that for referees in the WCBA, NBL, and other leagues (*F* = 2.818, *p* < 0.05, *η*^2^ = 0.023). By contrast, frustration tolerance for referees in the CBA league was superior to that for referees in the WCBA, NBL, and other leagues (*F* = 4.386, *p* < 0.01, *η*^2^ = 0.035). There was no difference in coping style and resilience among the referees across the various leagues (Table 3).

**Table 3:** Psychological resilience, coping style, mood state, and frustration tolerance among referees across different leagues.

	Sum of squares	Degrees of freedom	Mean square	*F*
Coping style	142.786	3	47.595	0.507
Mood state	3622.366	3	1207.455	2.818^**^
Frustration tolerance	4282.52	3	1427.507	4.386^**^
Psychological resilience	1913.315	3	637.772	2.452

**p < 0.01. F: Ratio of mean square between groups to mean square within groups.

### Correlations among coping style, psychological resilience, mood state, and frustration tolerance

Correlation analysis was used to analyze the relationships among the psychological variables. The results showed that coping style was negatively correlated with psychological resilience but positively correlated with mood state and frustration tolerance. Mood state was negatively correlated with psychological resilience but positively correlated with frustration tolerance. Psychological resilience was not correlated with frustration tolerance (Table 4).

**Table 4:** Correlations among coping style, psychological resilience, mood state, and frustration tolerance.

	Mean	SD	1	2	3	4
Coping style	31.89	9.68	1			
Psychological resilience	68.8	16.23	−0.180^**^	1		
Mood state	110.69	20.86	0.267^**^	−0.412^**^	1	
Frustration tolerance	80.26	18.3	0.203^**^	−0.053	0.291^**^	1

**p < 0.01. SD, standard deviation; 1: Coping style, 2: Psychological resilience, 3: Mood state, 4: Frustration tolerance.

### Regression analysis for coping style, mood state, age, referee grade, referee type, and referee period

We assessed the predictive effect of the independent variables on the dependent variables. On the basis of previous studies, we included age, referee grade, referee type, and referee period as control variables. For the control variables, the results of hierarchical regression showed that coping styles significantly and positively predicted mood state (*β* = 0.568, *p* < 0.001). The higher the level of coping style, the more stable was the mood state. However, only when age was used as a control variable was the result statistically significant, indicating that age was an important control factor for coping style to affect mood state (Table 5).

**Table 5:** Hierarchical regression results.

Variable	Model 1		Model 2		Model 3		Model 4		Model 5	
	E	STE	E	STE	E	STE	E	STE	E	STE
Intercept	92.31		106.5		95.42		94.68		93.04	
Coping style	0.576	0.267	0.585	0.271	0.573	0.266	0.568	0.263	0.568	0.263
Age			−0.39	−0.13	−0.39	−0.13	−0.36	−0.12	−0.45	−0.14
Referee grade					6.004	0.078	4.226	0.055	5.048	0.066
Referee type							1.082	0.059	1.538	0.084
Referee period									0.287	0.063
*R^2^*	0.071		0.088		0.094		0.097		0.099	
*F*	27.823^***^		17.328^*^		12.399		9.587		7.864	
*ΔR^2^*	0.071		0.016		0.006		0.003		0.002	
*ΔF*	27.823		6.416		2.408		1.137		0.974	

E, coefficient; STE, standardized coefficient. *p < 0.05; ***p < 0.001.

### Intermediary roles of frustration tolerance and psychological resilience

**Figure 2:**
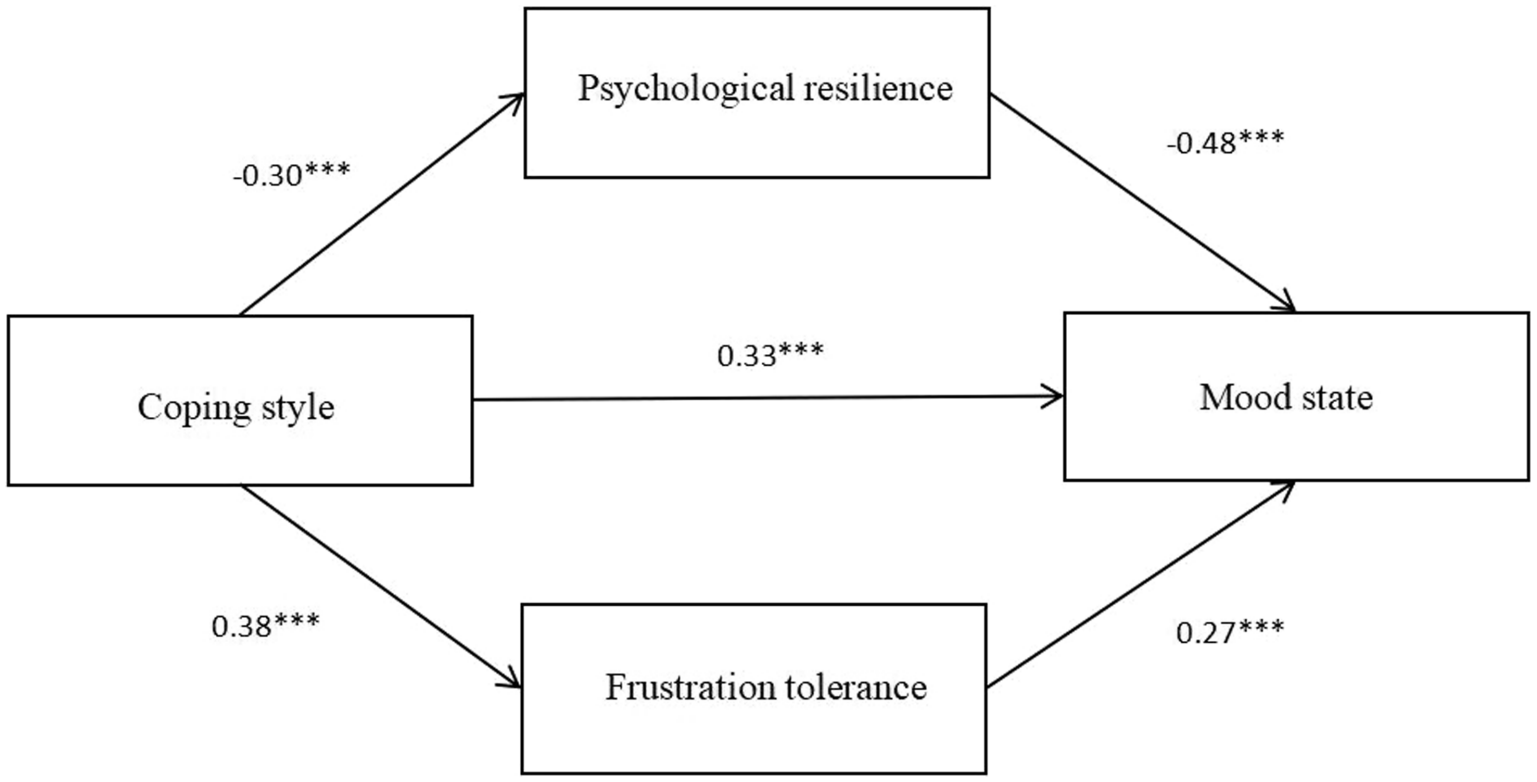
Parallel mediation model of coping style and mood state. ***p < 0.001.

We used the SPSS macro program Process compiled by Hayes and selected Model 4 in Templates to analyze the parallel mediation model. Coping style was the independent variable, psychological resilience and frustration tolerance were intermediary variables, and mood state was a dependent variable. The results showed that coping style had a negative predictive effect on psychological resilience (*β* = −0.30, *p* < 0.01), a positive predictive effect on frustration tolerance (*β* = 0.38, *p* < 0.01), and a positive predictive effect on mood state (*β* = 0.33, *p* < 0.01). In addition, psychological resilience had a negative predictive effect on mood state (*β* = −0.48, *p* < 0.01), and frustration tolerance had a positive predictive effect on mood state (*β* = 0.27, *p* < 0.01) (Table 6; Figure 2).

**Table 6:** Comparison of the statistical significance of path coefficients.

Path	β	SE	Bias-corrected 95% CI	
			Lower limit	Upper limit
Coping style → Psychological resilience	−0.3	0.087	−0.473	−0.132
Coping style → Frustration tolerance	0.38	0.097	0.192	0.575
Psychological resilience → Mood state	−0.48	0.059	−0.594	−0.362
Frustration tolerance → Mood state	0.27	0.053	0.171	0.378
Coping style → Mood state	0.33	0.101	0.128	0.526

β, standardized regression coefficient; SE, standardized error.

Statistical assessment of the non-standardized effect value corresponding to the action path of coping style influencing mood state indicated that the bootstrap 95% confidence interval of the total indirect and direct effects of coping style and mood state did not contain zero; Thus, the coping style of the referees significantly predicted their mood state (the effect value was 0.33, accounting for 56.9% of the total effect). This result was consistent with our first hypothesis. Moreover, the two intermediary variables psychological resilience and frustration tolerance have intermediary effects between coping style and mood state. The parallel mediation consisted of two indirect effects: (1) an indirect effect was produced by the path of coping style → psychological resilience → mood state. Its bootstrap confidence interval did not contain zero, indicating that psychological resilience had a significant mediating effect between coping style and mood state (the effect value was 0.14, accounting for 24.2% of the total effect). This result was consistent with our second hypothesis. (2) Another indirect effect was produced by the path of coping style → frustration tolerance → mood state. Its bootstrap confidence interval did not contain zero, indicating that frustration tolerance had a significant intermediary effect between coping style and mood state (the effect value was 0.11, accounting for 18.9% of the total effect). This result was consistent with our third hypothesis. The intermediary role of psychological resilience was greater than that of frustration tolerance (Table 7).

**Table 7:** Mediation effects and quantity tests.

Path	Effect value	Effect size	Bias-corrected 95% CI	
			Lower limit	Upper limit
Coping style → Psychological resilience → Mood state	0.14	24.20%	0.038	0.257
Coping style → Frustration tolerance→ Mood state	0.11	18.90%	0.352	0.199
Indirect effect	0.25	43.10%	0.110	0.395
Direct effect	0.33	56.90%	0.128	0.526
Total effect	0.58	100%	0.361	0.791

## Discussion

### Relationship between coping style and mood state of high-level basketball referees

The results of this cross-sectional survey study showed that the coping style of basketball referees positively predicted their mood state, which was consistent with our first hypothesis and with previous research results (Kardum and Hudek-Knežević, 1996). Whether an individual has psychological problems with negative emotions, such as depression and anxiety, after encountering a stressful event depends mainly on two aspects: the attributes of the event itself and the individual’s psychological susceptibility (Taylor et al., 1990). Psychological susceptibility includes having a negative cognitive tendency and cognitive process deviations from the norm, which emphasizes that an individual’s susceptibility quality is activated in a specific environment. Among susceptibility qualities, coping style receives the most interest. Relevant research shows that when individuals encounter a stressful event, if they cannot effectively cope with the pressure brought about by a related event, they are prone to have negative emotions, such as depression and anxiety (Dyrbye et al., 2006; Min et al., 2013). Job burnout and negative emotions based on the individual’s thoughts, emotions, behaviors, and personality characteristics reflect the level of their psychological flexibility. Individuals are affected by experience and other factors. Even when stressors are similar, an individual may use different coping strategies to deal with stress cognitive reappraisal at different times. During basketball matches, referees are typically in a highly stressed state. Having a positive coping styles helps referees calmly face various disturbances in the game, ensure the stability of his or her state of mind, and thus facilitate the orderly progress of the game. When basketball referees are facing pressure situations and self-perception decisions, their coping style may enable them to make intentional or unintentional attempts to adapt to the high-pressure environment or situation. Influenced by past experience, positive adaptation may help individuals avoid psychological crisis. Therefore, basketball referees with good coping styles can maintain a good state of mind to a certain extent and ensure the fairness and justice of the game.

### The mediating effects of frustration tolerance and psychological resilience among high-level basketball referees

This study found that the influence of coping styles on the mood states of high-level basketball referees was mainly realized through frustration tolerance and psychological resilience, both of which played an intermediary role between coping styles and mood states. Research shows that most basketball referees believe that a good pre-match meeting and communication with coaches, players, and peers effectively relieves their own psychological pressure while they are refereeing and that a good pre-match meeting and communication are also effective ways to deal with emergencies on the court (Wang, 2011). The frustration of basketball referees mainly comes from interference of family, life, work, coaches, players, fans, and spectators as well as the pressure of public opinion (Warner et al., 2013; Ridinger et al., 2017; Webb et al., 2020; Tingle et al., 2021). These setbacks require basketball referees to have better setback tolerance. If setback tolerance is low, it will have a negative psychological impact on the referees, which may easily lead to their unfairness on the court. Studies have found that individuals who show more positive attitudes, coping styles, and tenacity will also have higher resilience to setbacks (Si et al., 2022). Adopting a positive coping style may offset or avoid the negative impacts of setbacks or may even turn setbacks into favorable factors. Adopting a negative coping style will not eliminate the negative impact of setbacks but may actually strengthen their impact (Chen, 2008). In addition, Massey et al. (2009) found that individuals with lower anti-frustration abilities experience a stronger sense of frustration, lower sense of happiness, and higher negative emotions (Zhou et al., 2020). By contrast, the stronger the anti-frustration ability of adolescents, the less depressive symptoms they have and the more positive emotions they experience, thus improving their mood and their state of mind. In addition to this, the sense of community between referees could confirm the findings of this study, which is consistent with previous research (Kim et al., 2022). In the present study, frustration tolerance scores were high, indicating that basketball referees had a good frustration tolerance to adjust a negative state of mind. Good setback tolerance helps referees develop a strong will and maintain a positive state of mind, enabling them to have good adaptability during matches and to avoid anger and trouble caused by negative states of mind. Therefore, frustration tolerance plays a mediating role in the influence of coping styles on the state of mind in high-level basketball referees.

Increased individual resilience is due to the use of problem-solving and help-seeking coping styles, with a decreased use of fantasy and patience-coping styles. Problem-solving and help-seeking coping styles may be important protective factors in promoting the development of resilience, and a good emotional state is a positive influence on psychological resilience and coping styles. Emotioncy is a blend of the terms emotion and frequency and is commonly defined as sense-induced emotions that can relativize cognition. According to emotioncy, “individuals can be exvolved (hearing and seeing something) and involved (direct experience of something)” (Pishghadam et al., 2016). The level of emotioncy affects resilience and coping strategies. Depending on whether individuals are exvolved or involved in something changes their degree of resilience. The higher the level of emotioncy, the more probable emotioncy might be (Pishghadam et al., 2016). Individuals with strong coping styles continually strengthen their cognitive self-regulation and evaluation when dealing with negative events so as to adopt a positive perspective when facing diseases, establish a self-protection mechanism, and enhance internal anti-stress factors and psychological resilience (Friedberg and Malefakis, 2018). Sun et al. (2018) reported that the stronger an individual’s ability to resist pressure and adversity, the more stable and even positive the psychology in a disease period. State of mind contains positive psychological resources that are crucial for successful experiences and mental health, while resilience is related to a positive and optimistic state of mind, effective coping strategies, and positive results in education and mental health. The relationship between resilience and mood state can be understood through common genetic factors or non-common environmental factors. Riolli et al. (2010) found that state of mind is closely related to psychological resilience, and a positive state of mind better predicts psychological adjustment. In the face of adversity, individuals identify stressful stimuli, mobilize their own psychological resources that can cope with the stimuli, generate emotions, enter the stress process through cognitive evaluation, and finally reach an adaptive level of behavior (Min et al., 2013). Thus, psychological resilience is a mediating variable that indirectly predicts individual mood state.

As already mentioned, psychological resilience and frustration tolerance have some similarities. Individuals with high psychological resilience may reduce psychological distress caused by frustrating events through their flexible adjustment abilities. The study of psychological resilience originates from a study of children in adversity (experiencing setbacks). Researchers believed until the 1980s that adversity was disadvantageous to the development of children, with development following a linear model of adversity (frustration) leading to pressure, leading to maladjustment (Zhou and Cai, 2013). In the 1980s, researchers found that the model of development for children in adversity was not straight but curved: in the face of different setbacks, some children’s development was greatly limited, whereas other children’s development was very good, even beyond the normal level. Garmezy (1993) stated that people with high levels of resilience maintain strong competitiveness and adaptability in setbacks and can recover from setbacks without being defeated. Psychological resilience is a type of tolerance in personality traits, and tolerance belongs to psychological resilience in personality structure. Therefore, psychological resilience and frustration tolerance play parallel mediating roles in coping styles affecting mood states. The emo-sensory load may be an important variable in this study, just as the participants’ emotional sensory load also contribute to coping strategies, but this was not explored in the present study (Pishghadam and Shayesteh, 2017; Akbari and Pishghadam, 2022; Naji et al., 2022; Pishghadam et al., 2022). Individuals mostly do not pay attention to the emotional load of their own experiences, an issue that has been investigated in other studies (Pishghadam et al., 2019; Chang and Sun, 2021). Thus, our findings suggest that when training high-level basketball referees, increasing the psychological indicators related to the coping styles and psychological resilience of high-level basketball referees can avoid their large emotional fluctuations and improve their accuracy in judging when facing unexpected events on the court.

## Study limitations

This was a cross-sectional study and thus has limitations typical to that type of study. In the future, long-term follow-up studies should be conducted to confirm and extend our findings. Based on previous relevant research investigating coping style and mood state of high-level basketball referees in China, this study explored predictive results, analyzed mechanisms underlying the mood state of referees, and provided empirical research for exploring causality. However, the psychological variables assessed in this study do not include all the psychological activities of basketball referees and do not reveal causal relationships. Therefore, future research, should improve on our methods by using longitudinal studies to study participants over time and more deeply explore the factors that affect the psychological mechanisms of referees and that may reasonably explain any causal relationships. Although the variables selected in the present study are psychological reactions of referees in general, the psychological level of high-level basketball referees may be quite different. In the future, high-level referees should be interviewed first, and more comprehensive psychological indicators should be selected to make the results more compelling.

## Conclusion

The findings of this study showed that the coping style of high-level basketball referees in China was positively correlated with psychological resilience, frustration tolerance, and mood state, such that the higher the level of coping style was, the higher the level of psychological resilience, the higher the level of frustration tolerance, and the better the mood state. Psychological resilience and frustration tolerance played parallel intermediary roles between coping style and mood state of these basketball referees. Coping style had an indirect impact on mood state through psychological resilience and an indirect impact on mood state through frustration tolerance, with the intermediary effect of psychological resilience playing a greater role than the intermediary effect of frustration tolerance. These findings suggest that when training high-level basketball referees, increasing the psychological indicators related to the coping styles and psychological resilience of high-level basketball referees can avoid their large emotional fluctuations and improve their accuracy in judging when facing unexpected events on the court.

## Data availability statement

The original contributions presented in the study are included in the article/Supplementary material, further inquiries can be directed to the corresponding author.

## Ethics statement

The studies involving human participants were reviewed and approved by Ethics Committee of Yangzhou University Medicine College. The patients/participants provided their written informed consent to participate in this study.

## Author contributions

QW: conception, design of the study, and writing. WW: data collection, date organization, and writing. HH: data analysis. BW: data organization and formatting. All authors contributed to the article and approved the submitted version.

## Funding

This research was funded by 2022 Ministry of Education Humanities and Social Sciences Research Project (Project number: 22YJE890001).

## Conflict of interest

The authors declare that the research was conducted in the absence of any commercial or financial relationships that could be construed as a potential conflict of interest.

## Publisher’s note

All claims expressed in this article are solely those of the authors and do not necessarily represent those of their affiliated organizations, or those of the publisher, the editors and the reviewers. Any product that may be evaluated in this article, or claim that may be made by its manufacturer, is not guaranteed or endorsed by the publisher.

## References

[ref1] Akbari M. Pishghadam R. (2022). Developing new software to analyze the emosensory load of language. BCT 1, 1–13. doi: 10.56632/bct.2022.1101

[ref2] Anshel M. H. Sutarso T. Ekmekci R. Saraswati I. W. (2014). A model linking sources of stress to approach and avoidance coping styles of Turkish basketball referees. J. Sports Sci. 32, 116–128. doi: 10.1080/02640414.2013.816762, PMID: 24015999

[ref3] Brandão M. R. F. Serpa S. Rosado A. Weinberg R. (2014). Psychometric properties of the burnout inventory for referees. Motriz Revista de Educação Física 20, 374–383. doi: 10.1590/s1980-65742014000400003

[ref4] Chang S. Z. Sun Y. L. (2021). Influence of cognitive load and emotion on framing effect in risk decision-making of basketball players. J. Tianjin Univ. Sport 36, 569–573. doi: 10.13297/j.cnki.issn1005-0000.2021.05.011

[ref5] Chen W. (2008). Eliminate teachers' psychological frustration and maintain a good teaching mood. Modern Educ. Sci. 2, 22–23. doi: 10.3969/j.issn.1005-5843-B.2008.02.009

[ref6] Connor K. M. Davidson J. R. T. (2003). Development of a new resilience scale: the Connor-Davidson resilience scale (CD-RISC). Depress. Anxiety 18, 76–82. doi: 10.1002/da.10113 12964174

[ref7] Dyrbye L. N. Thomas M. R. Shanafelt T. D. (2006). Systematic review of depression, anxiety, and other indicators of psychological distress among US and Canadian medical students. Acad. Med. 81, 354–373. doi: 10.1097/00001888-200604000-00009, PMID: 16565188

[ref8] Friborg O. Barlaug D. Martinussen M. Rosenvinge J. H. Hjemdal O. (2005). Resilience in relation to personality and intelligence. Int. J. Methods Psychiatr. Res. 14, 29–42. doi: 10.1002/mpr.15, PMID: 16097398 PMC6878482

[ref9] Friedberg A. Malefakis D. (2018). Resilience, trauma, and coping. Psychodyn. Psychiatry 46, 81–113. doi: 10.1521/pdps.2018.46.1.81 29480784

[ref10] Garmezy N. (1993). Children in poverty: resilience despite risk. Psychiatry 56, 127–136. doi: 10.1080/00332747.1993.11024627 8488208

[ref11] Han F. L. Wang Q. L. (2022). Positive and negative mood states mediated the effects of psychological resilience on emotional stability among high school students during the COVID-19 pandemic. Front. Psychol. 13:7669. doi: 10.3389/fpsyg.2022.967669, PMID: 36046405 PMC9421361

[ref12] He P. Chen E. (2021). Developing tenacity: a new proposition for postgraduate education in the risk society. J. Grad. Educ. 3, 19–25. doi: 10.19834/j.cnki.yjsjy2011.2021.03.04

[ref13] Hu Z. J. (2010). The principle and method of estimating the statistical power and effect size when make Z test. Psychol. Explor. 30, 68–73. doi: 10.3969/j.issn.1003-5184.2010.01.014

[ref14] Kardum I. Hudek-Knežević J. (1996). The relationship between Eysenck's personality traits, coping styles and moods. Pers. Individ. Differ. 20, 341–350. doi: 10.1016/0191-8869(95)00182-4

[ref15] Kim M. Kim H. S. Simmond A. Warner S. (2022). Strengthening referees’ psychological well-being through engagement and authenticity. Sport Manag. Rev. 25, 254–274. doi: 10.1080/14413523.2021.1930952

[ref16] Li F. Li J. W. (2014). The effect of Normal Students' resilience, coping style on mental health. Chinese J. Health Psychol. 12, 1891–1893. doi: 10.13342/j.cnki.cjhp.2014.12.051

[ref17] Li Q. Yu K. Xie J. Chen Y. Zhao X. Liang C.. (2020). The influence of anti frustration psychological ability of higher vocational students on academic frustration: the mediation of Core literacy and coping style. Chin. J. Health Psychol. 28, 918–924. doi: 10.13342/j.cnki.cjhp.2020.06.027

[ref18] Lin X. (2008). Analysis and measures of basketball Referee's mental control on court. Bulletin Sport Sci. Technol. 16:47-48,71. doi: 10.3969/j.issn.1005-0256.2008.04.024

[ref19] Luo L. Zhou T. (2015). The relationship between gratitude and subjective well-being for middle-school students: the mediation role of anti-frustration ability and social support. Psychol. Dev. Educ. 31, 467–474. doi: 10.16187/j.cnki.issn1001-4918.2015.04.11

[ref20] Massey E. K. Garnefski N. Gebhardt W. A. (2009). Goal frustration, coping and well-being in the context of adolescent headache: a self-regulation approach. Eur. J. Pain 13, 977–984. doi: 10.1016/j.ejpain.2008.11.012, PMID: 19119033

[ref21] Min J.-A. Yu J. J. Lee C.-U. Chae J.-H. (2013). Cognitive emotion regulation strategies contributing to resilience in patients with depression and/or anxiety disorders. Compr. Psychiatry 54, 1190–1197. doi: 10.1016/j.comppsych.2013.05.008 23806709

[ref22] Naji E. Makiabadi H. Zabetipour M. Abbasnejad H. Firoozian Pooresfehani A. Shayesteh S. (2022). Emo-sensory communication, emo-sensoryn intelligence and gender. J. Bus. Commun. Technol. 1, 54–66. doi: 10.56632/bct.2022.1206

[ref23] Niu G. Hao E. Sun X. Zhou Z. (2013). Negative life Events' impact on depression among college students: the mediating effect of coping and the moderating effect of gender. Chin. J. Clin. Psych. 21, 1022–1025. doi: 10.16128/j.cnki.1005-3611.2013.06.016

[ref24] Onwukwe Y. U. (2010). The Relationship Between Positive Emotions and Psychological Resilience in Persons Experiencing Traumatic Crisis: A Quantitative Approach Capella University. Minneapolis: Capella University ProQuest Dissertations Publishing.

[ref25] Pishghadam R. Al Abdwani T. Kolahi Ahari M. Hasanzadeh S. Shayesteh S. (2022). Introducing metapathy as a movement beyond empathy: a case of socioeconomic status. Int. J. Soc. Cult. Lang. 10, 35–49. doi: 10.22034/ijscl.2022.252360

[ref26] Pishghadam R. Jajarmi H. Shayesteh S. (2016). Conceptualizing sensory relativism in light of Emotioncy: a movement beyond linguistic relativism. J. Soc. Cult. Lang. 4, 11–21.

[ref27] Pishghadam R. Makiabadi H. Shayesteh S. Zeynali S. (2019). Unveiling the passive aspect of motivation: insights from English language teachers’ habitus. Int. J. Soc. Cult. Lang. 7, 15–26.

[ref28] Pishghadam R. Shayesteh S. (2017). Emo-sensory expression at the crossroads of emotion, sense, and language: a case of color-emotion associations. Int. J. Soc. Cult. Lang. 5, 15–25.

[ref29] Pizzera A. Laborde S. Lahey J. Wahl P. (2022). Nfluence of physical and psychological stress on decision-making performance of soccer referees. J. Sports Sci. 40, 2037–2046. doi: 10.1080/02640414.2022.2127516, PMID: 36175198

[ref30] Ridinger L. L. Kim K. R. Warner S. Tingle J. K. (2017). Development of the referee retention scale. J. Sport Manag. 31, 514–527. doi: 10.1123/jsm.2017-0065

[ref31] Riolli L. Savicki V. Spain E. (2010). Positive emotions in traumatic conditions: mediation of appraisal and mood for military personnel. Mil. Psychol. 22, 207–223. doi: 10.1080/08995601003638975

[ref32] Si T. Wu M. Zhang X. (2022). Impact of college students life vision on core literacy: the mediating role of life meaning and cell phone dependence. China J. Health 30, 106–112. doi: 10.13342/j.cnki.cjhp.2022.01.022

[ref33] Sun M. Bi R. Wang Y. Hao Y. (2018). Relationship among optimism, pessimism and well-being in college students. Chin. Ment. Health J. 32, 615–619. doi: 10.3969/j.issn.1000-6729.2018.07.014

[ref34] Tao Y. (2016). Chinese basketball Referee's ability of control at different matches. J. Wuhan Sports Univ. 50, 96–100. doi: 10.15930/j.cnki.wtxb.2016.11.016

[ref35] Taylor A. H. Daniel J. V. Leith L. Burke R. J. (1990). Perceived stress, psychological burnout and paths to turnover intentions among sport officials. J. Appl. Sport Psychol. 2, 84–97. doi: 10.1080/10413209008406422

[ref36] Tingle J. K. Jacobs B. L. Ridinger L. L. Warner S. (2021). Female sports officials and mental health: the overlooked problem. J. Sport Manag. 36, 383–393. doi: 10.1123/jsm.2020-0443

[ref37] Wang Z. (2011). Research on temperament and coping style of excellent basketball referees in China. J. Mudanjing Teach. Coll. 2, 46–47. doi: 10.3969/j.issn.1003-6180.2011.02.022

[ref38] Wang Y. (2017). Impact of emotion and expertise on football referees' foul decision making. J. Wuhan Inst. Phys. Educ. 51, 96–100. doi: 10.15930/j.cnki.wtxb.2017.01.016

[ref39] Wang W. Jiang Y. (2018). Effects of emotional regulation strategy and resilience on the cognitive control of football players. J. Tianjin Univ. Sport 33, 52–57. doi: 10.13297/j.cnki.issn1005-0000.2018.01.009

[ref40] Wang J. Wang X. Li L. (2014). Revised Chinese version of frustration tolerance questionnaire (FDS). Theory Res. 2, 56–57. doi: 10.3969/j.issn.1002-2589.2014.02.025

[ref41] Wang L. Yang P. Tao B. Yan J. (2021). The relationship between self-control and emotional stability of high-level basketball referees in China: the chain mediating effect of mood state and mental resilience. Sports Sci. 42, 86–95. doi: 10.13598/j.issn1004-4590.2021.06.013

[ref42] Warner S. Tingle J. Kellett P. (2013). Officiating attrition: considering the experiences of referees from a sport development perspective. J. Sport Manag. 27, 316–328. doi: 10.1123/jsm.27.4.316

[ref43] Webb T. Dicks M. Thelwell R. van Der Kamp J. Rix-Lievre G. (2020). An analysis of soccer referee experiences in France and the Netherlands: abuse, conflict, and level of support. Sport Manag. Rev. 23, 52–65. doi: 10.1016/j.smr.2019.03.003

[ref44] Ye Y. Shen Y. (2002). A summary of the study of coping and ways of coping. J. Psychol. Sci. 6, 755–756. doi: 10.16719/j.cnki.1671-6981.2002.06.040

[ref45] Yu X. N. Zhang J. X. (2007). Factor analysis and psychometric evaluation of the Connor-Davidson resilience scale (CD-RISC) with Chinese people. Soc. Behav. Pers. 35, 19–30. doi: 10.2224/sbp.2007.35.1.19

[ref46] Zhang J. Liu T. Chen Y. Wang H. (2021). Effect of interpersonal sensitivity on mental health of rural college students with left-behind experience: multiple mediating effects of self-efficacy and emotion. J. HeBei United Univ. 23:303-308+315. doi: 10.19539/j.cnki.2095-2694.2021.04.010

[ref47] Zhang Y. Yan K. Wang J. (2005). A path analysis on life events, negative automatic thoughts, coping style and depression. Psychol. Dev. Educ. 21, 96–99. doi: 10.3969/j.issn.1001-4918.2005.01.018

[ref48] Zhou Y. Cai M. (2013). The relationship among positive emotions, resilience and frustration tolerance of college students. Acad. Explorat. 7, 149–152. doi: 10.3969/j.issn.1006-723X.2013.07.033

[ref49] Zhou T. Zhang Q. Wang C. (2020). The impact of social support on anti-frustration ability among middle school students: a serial mediation effect model. J. Southwest China Normal Univ. 45, 88–96. doi: 10.13718/j.cnki.xsxb.2020.06.014

[ref50] Zhu B. (1995). Brief introduction of POMS scale and its model for China. J. Tianjin Sports Inst. 1, 35–37.

